# Pathogen Identification in Suspected Cases of Pyogenic Spondylodiscitis

**DOI:** 10.3389/fcimb.2017.00060

**Published:** 2017-03-09

**Authors:** Ahmad Farajzadeh Sheikh, Azar D. Khosravi, Hamed Goodarzi, Roohangiz Nashibi, Alireaza Teimouri, Azim Motamedfar, Reza Ranjbar, Sara Afzalzadeh, Mehrandokht Cyrus, Mohammad Hashemzadeh

**Affiliations:** ^1^Infectious and Tropical Diseases Research Center, Health Research Institute, Ahvaz Jundishapur University of Medical SciencesAhvaz, Iran; ^2^Department of Microbiology, School of Medicine, Ahvaz Jundishapur University of Medical SciencesAhvaz, Iran; ^3^Molecular Biology Research Center, Baqiyatallah University of Medical ScienceTehran, Iran; ^4^Department of Infectious Diseases, Razi Teaching Hospital, Ahvaz Jundishapur University of Medical SciencesAhvaz, Iran; ^5^Department of Neurosurgery, Golestan Teaching Hospital, Ahvaz Jundishapur University of Medical SciencesAhvaz, Iran; ^6^Department of Neurosurgery, Tehran Medical Science Branch, Islamic Azad UniversityTehran, Iran; ^7^Department of Radiology, Razi Teaching Hospital, Ahvaz Jundishapur University of Medical SciencesAhvaz, Iran

**Keywords:** MRI, pyogenic spondylodiscitis, biopsy, polymerase chain reaction, bacterial agents

## Abstract

Pyogenic spinal infection continues to represent a worldwide problem. In approximately one-third of patients with pyogenic spondylodiscitis, the infectious agent is never identified. Of the cases that lead to organismal identification, bacteria are more commonly isolated from the spine rather than fungi and parasites. This study applied universal prokaryotic *16S rRNA* PCR as a rapid diagnostic tool for the detection of bacterial agents in specimens from patients suspected of pyogenic spondylodiscitis. Gram and Ziehl-Neelsen staining were used as a preliminary screening measure for microbiologic evaluation of patient samples. PCR amplification targeting *16S rRNA* gene was performed on DNA extracted from 57 cases including specimens from epidural abscesses, vertebral, and disc biopsies. Positive samples were directly sequenced. MRI findings demonstrated that disc destruction and inflammation were the major imaging features of suspected pyogenic spondylodiscitis cases, as 44 cases showed such features. The most common site of infection was the lumbar spine (66.7%), followed by thoracic spine (19%), the sacroiliac joint (9.5%), and lumbar-thoracic spine (4.8%) regions. A total of 21 samples amplified the *16S rRNA*-PCR product. Sanger sequencing of the PCR products identified the following bacteriological agents: *Mycobacterium tuberculosis* (*n* = 9; 42.9%), *Staphylococcus aureus* (*n* = 6; 28.5%), *Mycobacterium abscessus* (*n* = 5; 23.8%), and *Mycobacterium chelonae* (*n* = 1; 4.8%). 36 samples displayed no visible *16S rRNA* PCR signal, which suggested that non-bacterial infectious agents (e.g., fungi) or non-infectious processes (e.g., inflammatory, or neoplastic) may be responsible for some of these cases. The L3–L4 site (23.8%) was the most frequent site of infection. Single disc/vertebral infection were observed in 9 patients (42.85%), while 12 patients (57.15%) had 2 infected adjacent vertebrae. Elevated erythrocyte sedimentation rate (ESR) and C-reactive protein (CRP) inflammatory markers were noted in majority of the patients. In conclusion, microbiological methods and MRI findings are vital components for the proper diagnosis of pyogenic spondylodiscitis. Our findings suggest that molecular methods such as clinical application of *16S rRNA* PCR and sequencing may be useful as adjunctive diagnostic tools for pyogenic spondylodiscitis. The rapid turnaround time of *16S rRNA* PCR and sequencing submission and results can potentially decrease the time to diagnosis and improve the therapeutic management and outcome of these infections. Although *S. aureus* and *M. tuberculosis* were the most common causes of pyogenic spinal infections in this study, other infectious agents and non-infectious etiologies should be considered. Based on study results, we advise that antibiotic therapy should be initiated after a definitive etiological diagnosis.

## Introduction

Pyogenic spinal infection continues to be a worldwide problem. Pyogenic spinal infection represents a rare but broad spectrum of diseases such as pyogenic spondylitis, spondylodiscitis (vertebral osteomyelitis), septic discitis, and epidural abscess. Infection is often spontaneous and hematogenous in origin but can also occur after invasive spinal surgery (Lecouvet et al., 2004; Duarte and Vaccaro, 2013). There is still inadequate knowledge about all risk factors, clinical features, diagnostic methods, and appropriate treatment for adults with spondylodiscitis (Cebrian Parra et al., 2012; Lopez-Duran Stern and Leon Serrano, 2012). Known significant predisposing factors that could contribute to spinal infections include previous spine surgery, a distant infectious focus, diabetes mellitus, advanced age, intravenous drug use, HIV infection, immunosuppression, oncologic history, and renal failure (Duarte and Vaccaro, 2013). Pyogenic spondylodiscitis diagnosis is typically made on the basis of clinical symptoms, serological and hematological laboratory data, radiological findings, and employing other laboratory procedures on tissue samples including microbiological culture, histology, and molecular analyses. Although clinical findings and serological laboratory data are sensitive, they lack diagnostic specificity for pyogenic spondylodiscitis. Radiological findings, especially magnetic resonance imaging (MRI), may show specific changes in some pyogenic spondylodiscitis cases, but provide no information about the etiological agent (Sobottke et al., 2008; Zimmerli, 2010). Since the spectrum of etiologic agents is diverse, the definitive diagnosis of the causative agent is through microbiologic culture or other rapid identification tests. Exact determination of the agent is required to optimize effective therapy, which improves the outcome of the disease in most patients (Gouliouris et al., 2010). Moreover, well-timed and appropriate antimicrobial therapy is essential to prevent prolonged suffering and severe complications (Meredith et al., 2012). However, microbiological diagnosis may fail due to antibiotic treatment before tissue sampling or difficulty in culturing the causative agent (Chelsom and Solberg, 1998; Butler et al., 2006). In fact, the etiological agent is never identified in approximately one-third of pyogenic spondylodiscitis cases (Govender, 2005). Since appropriate antibiotic selection is often difficult, broad spectrum antibiotic are usually administered in culture-negative cases. Inappropriate antibiotic use can result in prolonged hospital stays and increased costs, but it can also have adverse consequences on the patient's prognosis.

Diagnosis of the disease as well as identification of its causative agent is still in its early stages. The utility of molecular methods for the detection of pathogenic agents in pyogenic spondylodiscitis has recently been enhanced by the use of various rapid PCR techniques that have increased sensitivity compared to culture and clinical observations (Kobayashi et al., 2005, 2009).

Of the cases that do lead to organismal identification, bacteria are more commonly isolated from the spine rather than fungi or parasites. *S. aureus, M. tuberculosis, Brucella* spp., Gram-negative and anaerobic bacteria are among the most common pathogens recovered from pyogenic discitis and spinal infections (Lecouvet et al., 2004). Symptoms related to these infections are typically regarded as an outcome of recurring disc projection and an unsatisfactory operation, leading to treatment difficulty and permanent damage (Hamdan, 2012). Diagnosis is often difficult, as no specific signs or symptoms are present. Additionally, other possibilities such as rarity of the disease which often causes delays in diagnosis after the initial onset of symptoms and the high frequency of low back pain in the general population should also be considered (Glaudemans et al., 2012).

In this work, we evaluated Erythrocyte Sedimentation Rate (ESR) and C- reactive protein (CRP) as sensitive markers, and MRI as preliminary evaluation tools for spinal infections. Causative agents were then confirmed by *16S rRNA* sequence analysis.

## Materials and methods

### Clinical specimen's preparation

This study included a total of 57 suspected spondylodiscitis patients referred by 3 centers in Ahvaz, Iran (Arvand, Golestan and Razi teaching hospitals) from January 2014 to June 2015. Vulnerable adults were not included in this study. Enrolled patients included 40 men and 17 women, with mean age of 49 years (ranged from 24 to 72 years), 5 patients had a neurosurgical operation and 52 suspected cases of pyogenic spondylodiscitis with spontaneous discitis. Inclusion criteria were as follows: (1) clinical symptoms suspected of discitis (back pain on physical activities and no alleviation of pain with rest); (2) MRI features compatible with disc space infection and infectious spondylodiscitis (vertebral end plate destruction, disc inflammation, and presence of necrosis or pus in the disc space and paraspinal soft tissues and epidural spaces); and (3) Cardinal and para-clinical features including elevated ESR (reference range 0–20 mm/h), elevated CRP (reference range 0–10 mg/l), and presence of inflammatory signs such as fever (above 38°C) (Hamdan, 2012; Meredith et al., 2012; Lee, 2014).

For bacteriological analysis, samples were taken by puncture or biopsy from affected area of disc space, epidural abscess, or vertebral body by radiologist or neurosurgeon under sterile conditions (Lecouvet et al., 2004). Particular attention was paid to the following variables in patient medical history: Gender, age, and date of onset of discitis, and history of conditions such as surgery, sepsis, and back pain.

### Microbiologic analyses

#### Isolation of bacteria

Each sample was divided into two equal portions for staining and molecular procedures. Gram and Ziehl-Neelsen staining were used as preliminary screening measures in patient samples. The remaining sample was stored at −70°C for *16S rRNA* PCR.

### Molecular identification of bacteria

This study applied universal prokaryotic *16S rRNA* PCR as a rapid diagnostic for the detection of bacterial agents in samples from patients suspected of pyogenic spondylodiscitis. PCR was performed under strict conditions. All pre- and post-PCR manipulations were performed in separate designated rooms with sterilized pipetting tools to prevent contamination of the samples with foreign DNA. Also, DNA-free tools were used for the pre-amplification steps and further irradiated by ultraviolet (UV) light to avoid causing false-positive results.

#### PCR assay

The genomic DNA was extracted by High Pure PCR Template preparation kit (Roche, Germany).

Sample DNA was PCR amplified for *16S rRNA* genes (1500 bp) using universal primers pA (5′-AGAGTTTGATCCTGGCTCAG-3′) and pI (5′-TGCACACA-GGCCACAAGGGA-3′) as described by Rogall et al. (1990). DNA amplification was performed in a thermocycler nexus gradient (Eppendorf, Germany), in a final volume of 25 μl containing 10x PCR buffer, 1.5 mM Mg Cl_2_, 10 mM dNTPs, 0.5 μM of each primer, 1.5 U super *Taq* polymerase and 5 μl of template DNA. The cycling parameters included: Initial denaturation at 95°C for 5 min, 35 cycles of denaturation at 95°C for 30 s, annealing at 65°C for 1 min, and extension at 72°C for 1 min and a further step of final extension at 72°C for 7 min. The amplified products were subjected to electrophoresis on 2% agarose gel, stained with 0.5 μg/μl ethidium bromide (Qiagen, Germany) and analyzed under UV light in a gel documentation system (Protein Simple, San Jose, CA, USA). A 100 bp DNA ladder was used as a size marker (Roche, Germany).

The amplified PCR products of *16S rRNA* gene for each isolate were purified with the Gene JET™ Gel Extraction Kit (Fermentas, Lithuania) according to manufacturer's instructions. The sequences of the products were determined using an ABI PRISM 7700 Sequence Detection System (Applied Biosystems, Foster City, Calif.) according to the standard protocol of the supplier.

#### Analysis of sequence data

The obtained sequences for each isolate were aligned separately and compared with all existing relevant sequences retrieved from GenBank database using MEGA6 program (Jeon et al., 2005; Tamura et al., 2007). Percentages of similarity between sequences of *16S rRNA* gene were determined by comparing sequences search to an in-house database of *16S rRNA* genes region sequences. One sample with positive PCR result was previously sequenced by the Bioneer Company, Korea (confirmed for *M. tuberculosis*) and used as positive control, while distilled water was used as a negative control.

### Statistical analysis

Comparison of differences for categorical data performed by the Chi-square with Fishers' exact test.

## Results

### Clinical presentation

Low back pain, muscle spasm, local tenderness, fever, limitation of motion, inability to bend, weight loss, and avoiding any movements were the initial clinical findings. None of the patients were immunocompromised, 5 patients had a neurosurgical operation and co-morbidity diseases were seen in 12 cases of 21 patients comprising 4 patients with diabetes mellitus (19%), 4 patients with tuberculosis (TB) (19%), and 2 patients with renal failure (9.5%). Paravertebral, paraspinal and epidural abscess were detected by MRI only in 5 patients (23.8%). None of the patients were under non-steroidal anti-inflammatory treatment, but 4 patients had received empiric intravenous antibiotic therapy against the most common bacterial agents for spondylodiscitis. Elevated ESR was a useful marker for the diagnosis, which was between 37 and 110 mm/h; also a raised CRP between 10 and 51 mg/l was a constant result. Mean WBC count was 9,514 cells/μL (range 4,400–19,300); 14 patients had lower than 10,000 cells/μL, while only 7 patients had over 10,000 cells/μL. The WBC count were not useful and not an important diagnostic criteria, due to its low sensitivity.

Of 9 spinal TB cases, 1 patient died because of chronic renal failure (CRF), and diabetes mellitus.

### MRI

**Figure 1:**
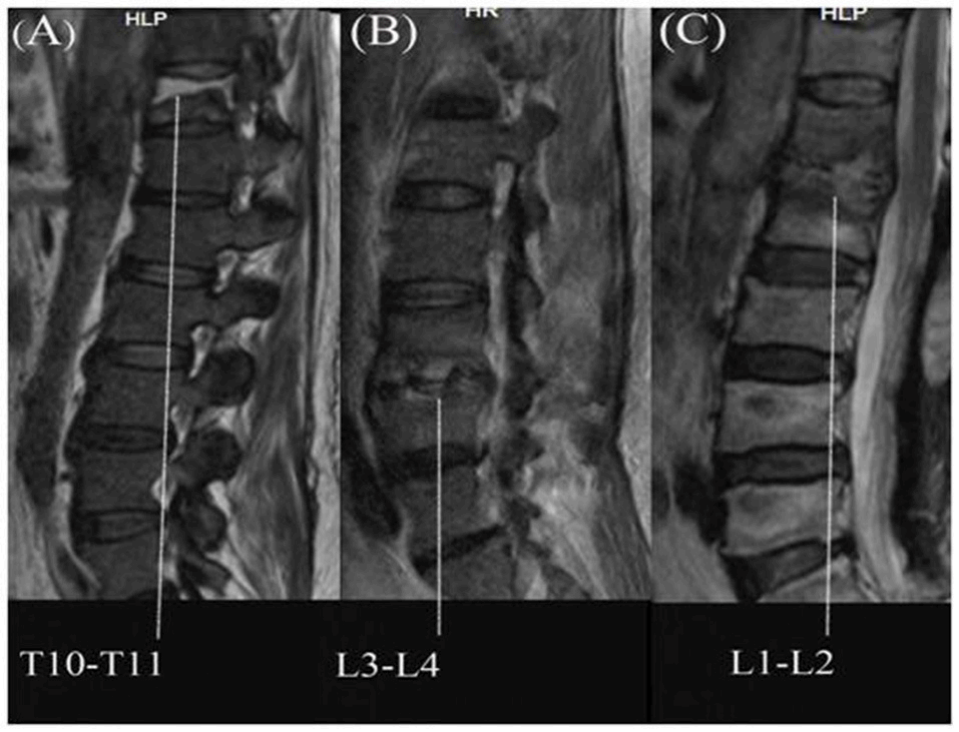
**Magnetic resonance imaging of infectious spondylitis; (A)** Tuberculous spondylitis; **(B)** Staphylococcal spondilitis; **(C)** NTM spondilitis.

**Figure 2:**
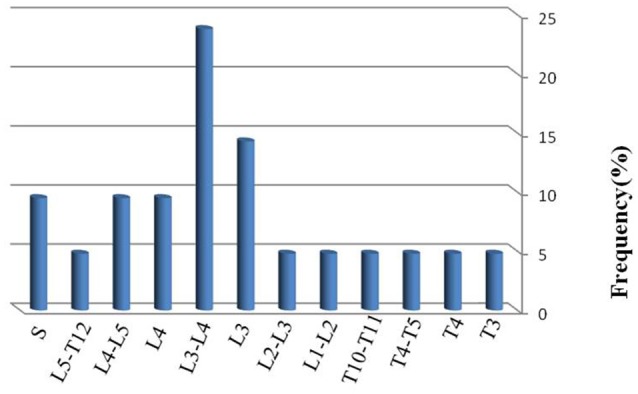
**Vertebral levels affected in 21 patients with spinal infection**. Only one level was affected in 9 patients. Two levels were affected in 12 patients.

MRI was done in all patients with very significant findings 44/57 (77.2%) and clearly demonstrated the presence and location of any abscess, vertebral body involvement with destruction and inflammation in several cases. Based on the MRI findings and PCR results, the organisms responsible for the spondylodiscitis infections were identified as *M. tuberculosis* (Figure 1A), *S. aureus* (Figure 1B), and *M. abscessus* (Figure 1C). MRI results demonstrated that disc destruction and inflammation were the major clinical features of suspected pyogenic spondylodiscitis cases. Among the 36 cases with negative PCR assay, vertebral body involvement wasn't detected in 13/36 (36.1%) by MRI. The most common site of infection was the lumbar spine (66.7%), followed by thoracic (19%), sacroiliac joint (9.5%), and lumbar-thoracic spine (4.8%) regions. The patients' data are summarized in Table 1. Figure 2 represents a summary of the location of the lesions identified by imaging among the patients with positive PCR products.

**Table 1:** **Clinical feature of 21 pyogenic spondylodiscitis patients confirmed by *16S rRNA* PCR**.

**NO**	**past medical history**	**Tuberculosis close contact**	**Previous Surgery**	**Direct microbiologic exam of stained smears**	**Clinical data**	**Site of Involvement**	**WBC^a^**	**ESR^b^Mm/h**	**CRP^c^Mg/dl**	**Organism**
5	PID^d^	–	–	Gram positive cocci	Fever and limitation of motion, Sacroiliac abscess	Sacroiliac	14,700	135	10	*S. aureus*
7	Renal failure	+	–	–	Hemiparesis and weight loss, epidural and paravertebral abscess	T4–T5	4,400	108	44	*M. tuberculosis*
8	Addicts	+	–	Acid fast bacilli	Low back pain	L3	7,600	102	–	*M. abscessus*
11	Diabetes, Renal failure	–	–	Acid fast bacilli	Low back pain	L4	12,800	87	11	*M. abscessus*
14	Pulmonary Tuberculosis	–	–	–	Low back pain	L3–L4	6,700	110	9	*M. tuberculosis*
15	Hypertension	+	–	–	Local tenderness	T3	8,900	45	–	*M. tuberculosis*
17	Diabetes melitus, metastatic lung cancer	–	–	–	Low back pain	L3	7,800	70	47	*M. abscessus*
21	–	–	+	Gram positive cocci	Fever and Low back pain	S1	10,400	78	51	*S. aureus*
23	Hypertension	–	–	–	Fever and Low back pain, AFB positive, drug hepatitis	L5–T12	11,600	99	39	*M. tuberculosis*
27	–	+	+	Gram positive cocci	Low back pain, Paravertebral abscess	L3–L4	7,900	98	15	*S. aureus*
28	–	–	+	Gram positive cocci	Low back pain, weight loss, fever	L3–L4	19,300	37	14	*S. aureus*
31	Extrapulmonary Tuberculosis, Rhinoplasty	–	–	–	Local tenderness	L3	8,500	38	–	*M. tuberculosis*
35	Diabetes melitus,IHD^e^, Pulmonary Tuberculosis	–	–	–	Low back pain	L3–L4	9,800	58	–	*M. tuberculosis*
36	Pulmonary Tuberculosis	–	–	Acid fast bacilli	Low back pain	L4–L5	8,500	78	11	*M. tuberculosis*
37	IHD	–	–	Gram positive cocci	Local tenderness	L2–L3	6,500	68	12	*S. aureus*
41	Diabetes	+	–	Acid fast bacilli	Low back pain	L4–L5	5,200	79	10	*M. abscessus*
44	–	+	–	–	Fever and cough and dyspnea, Paravertebral abscess, Lower thoracic abscess	T4	7,900	54	39	*M. tuberculosis*
45	Hypertension, Appendicitis, AKI^f^	–	+	–	Fever and hemiparesis and low back pain	L4	9,300	46	42	*S. aureus*
48		–	–	–	Fever and low back pain, Paraspinal abscess	L3–L4	10,400	77	25	*M. abscessus*
51	–	–	–	–	Fever and low back pain	L1–L2	11,900	98	28	*M.chelonea*
57	–	–	–	–	Low back pain, spasticity and limitation of motion	T10–T11	9,700	54	11	*M. tuberculosis*

### Microscopic analysis

By microscopic analysis, using Gram and Ziehl Neelsen staining, only 9 out of 57 samples were positive and diagnosed as bacterial agents (Gram positive cocci revealed in 5 samples and 4 samples were positive for acid fast bacilli).

### Molecular and sequencing analysis

In order to confirm the preliminary diagnosis of spondylodiscitis by radiological examination, *16S rRNA*-PCR based sequencing analysis was used, and a total of 21 samples were PCR positive. Based on sequences of the PCR products, three representative cases of spondylodiscitis with molecular confirmation of the causative agents are shown in Figures 1A–C. The nucleotide sequences were compared pairwise for similarity in BLAST search tool. Sanger sequencing of the *16S rRNA* gene identified the following bacteriological agents: *M. tuberculosis* (*n* = 9; 42.9%), *S. aureus* (*n* = 6; 28.5%), *M. abscessus* (*n* = 5; 23.8%), and *M. chelonae* (*n* = 1; 4.8%). 36 samples displayed no visible *16S rRNA* PCR signal, which suggests that non-bacterial infectious agents (e.g., fungi) or non-infectious processes (e.g., Inflammatory, or neoplastic) may be responsible for some of these cases. The L3–L4 site (23.8%) was the most frequent site of infection. Single disc/vertebral infection was observed in 9 patients (42.85%), while 12 patients (57.15%) had 2 infected adjacent vertebrae. Elevated ESR and CRP serological markers were noted in the all of patients with PCR positive products.

## Discussion

Pyogenic spondylodiscitis is identified usually by clinical symptoms, serological and hematological laboratory data, radiological findings, microbiological culture, histology, and molecular biology analyses. Culture is the gold standard method for the diagnosis of spinal infections, although it is time-consuming especially in case of slow-growing pathogens. In order to expedite the rapid diagnosis of spinal infection, molecular methods have been recently applied by the use of various PCR and real time PCR techniques (Choe et al., 2014).

In the present study concordant to other reports (Butler et al., 2006; Frangen et al., 2006; Tsiodras and Falagas, 2006), there was a 2–6 month delay between onset of the first symptoms and accurate diagnosis of spondylodiscitis, mainly due to the low specificity of clinical signs and symptoms, which can lead to tragic outcomes in some cases. The disease is usually observed in patients aged over 50 years, and in our study, 60.9% of the patients were older than 50 years with a mean age of 49 years. Spondylodiscitis may be associated with an epidural abscess or granulation tissue in 25–50% of cases (Turgut, 2008). In our study, 5 cases (23.8%) were associated with abscess that could be caused by either direct extension from spondylodiscitis or inoculation from an invasive spinal procedure. Based on a previous study, about 5% of patients with spinal epidural abscesses die, usually because of uncontrolled meningitis, sepsis or other underlying illnesses (Shioya et al., 2012). In our study, only 1 death (4.35%) occurred due to renal failure in a diabetic patient with spinal tuberculosis. In the present study, local back pain, fever, limitation of motion, and localized tenderness near the affected area were the most common symptoms in spondylodiscitis patients. Additionally, CRP and ESR also significantly rose in all patients. Although CRP and ESR are considered as non-specific parameters for inflammation, these are among the useful diagnostic and prognostic tests to support clinical diagnosis of spondylodiscitis, and are of value in the follow-up treatment. ESR, as an inflammatory index, is reported to be always high in spinal infection. The mean ESR was 84.5 mm/h in a Malaysian report (Dharmalingam, 2004), and 48 mm/h in an Iranian study conducted on a period 1971 and 1995 (Jabalameli and Ameri, 1998), while in the present study, the mean ESR was 70.4 mm/h, higher than that of the previous report from Iran. In contrast, the WBC count (reference range 4,000–10,000 cell/μL) was not a very useful inflammatory marker. The mean of WBC count was 9,514 cells/μL (4,400–19,300) and 14 patients had a WBC count lower than 10,000 cells/μL, while only 7 patients had over 10,000 cells/μL.

MRI is reported as the most sensitive and specific test for preliminary detection of spondylodiscitis (Meredith et al., 2012). In this work, radiologic findings were also compatible with disc space infection (confirmed by PCR technique). Although vertebral infection could be diagnosed by MRI, typical features such as disc space narrowing, end plates attrition, and calcification of the annulus around the affected disc usually become visible only in the late stage of the disease. Postoperative spinal infections are usually diagnosed by microbiologic methods (Gram and Ziehl neelsen staining, culture, etc). However, in some spinal infections such as tuberculous disc infection with associated non-specific clinical signs, definitive diagnosis can be difficult, which may cause delays in effective treatment.

Although the diagnosis of spondylodiscitis is commonly based on clinical data, recent introduction of molecular methods including PCR technique allows a more accurate diagnosis. Therefore, in this work, amplification by PCR was performed on *16S rDNA* universal target genes. Species identification relied on the amplicon sequencing and comparison with templates from Gen-Bank. The results from PCR method revealed the detection of causative bacteria in 21 out of 57 biopsy samples with *M. tuberculosis* as the major isolated organism (42.9%). The aim of this study was to identify potential bacterial causative agents in pyogenic spondylodiscitis, so for the remaining 36 PCR negative samples, alternative diagnoses are non-bacterial infections that could be related to fungi, protozoa, etc, as well as non-infectious etiologies (i.e., inflammatory and neoplastic). Among 9 PCR-positive cases of *M. tuberculosis*, 4 had past history of tuberculosis (TB), and another 4 had close contact with a TB patient. Spinal TB could result from a primary pulmonary infection or continuous contact with a TB source. In our study, only 5 samples were identified as Gram positive cocci on microscopic examination, which were later confirmed by PCR method and identified as *S. aureus*. Moreover, 4 samples with positive Ziehl Neelsen staining representing acid fast bacilli were later confirmed by PCR method as 3 cases of spinal infection involving non-tuberculous mycobacteria (NTM), and 1 case of spinal TB infection.

Extrapulmonary tuberculosis (EPTB) constitutes about 15–20% of all cases of TB, and although the exact incidence of bone TB is unknown, it is estimated that spinal TB accounts for 1–5% of all TB cases (Cebrian Parra et al., 2012). Bone TB is probably the most common EPTB, which could affect any part of the spine and skeletal system (Agrawal et al., 2010). TB is an endemic and prevalent infectious disease in Khuzestan, Iran, and in our study *M. tuberculosis* was also the most common cause of spinal infections (42.9%). EPTB infections are usually more common among AIDS patients, but in our study, none of the patients had HIV/AIDS. Instead, the 9 positive samples for *M. tuberculosis* were recovered from 3 cases (33.3%) with pulmonary TB, 1 case with spinal TB, 1 case with diabetes mellitus and 1 case with renal failure (each representing 11.11%). According to the literature, spinal TB mostly affects the thoracic spine and less often the lumbar spine (Hong et al., 2009). However, in the present study, thoracic and lumbar vertebrae were approximately involved equally by *M. tuberculosis*.

NTMs can be rarely involved in vertebral osteomyelitis and are frequently associated with surgical procedures as well as environmental contamination of microbiologic specimens. In a literature review (Petitjean et al., 2004) that was performed on spinal infections between 1965 and December 2003, *Mycobacterium avium* complex was the most frequent NTM encountered in these infections. However, other NTMs including *M. xenopi, M. fortuitum*, and *M. abscessus* were also detected (Petitjean et al., 2004). In the present study, *M. abscessus* with a rate of 28.6% was the most frequently detected NTM and half of the patients infected with this mycobacterium had diabetes as a potential predisposing factor.

In the present study, the lower lumbar vertebrae were most frequently involved in spondylitis patients, and the most frequent manifestations of spondylodiscitis were seen in lumbar region (66.7%), and less frequently in the thoracic area” (19%). An abscess (Paravertebral, spinal and epidural) was reported in 5 (23.8%) cases. In a study conducted by (Devkota et al., 2014), L4–L5 level was the most frequent site of pyogenic discitis, while in our study L3–L4 site represented the most common level of infection noticed in 5 cases (23.8%), which was similar to the findings reported by other investigators (Jabalameli and Ameri, 1998; Hadadi et al., 2010). Involvement of this site is believed to be due to the relatively large vertebral body and disc space. Additionally, in our study, multiple-level skip lesions (combinations) were seen in 2 cases and also, simultaneous involvement of 2 vertebrae was identified in 12 cases, whereas single vertebral involvement was seen in 9 cases.

In previous studies *S. aureus* was reported as the predominant pathogen for spondylodiscitis (Ross and Fleming, 1976; Collert, 1977), while our findings showed a higher detection rate of *M. tuberculosis*. Among 6 staphylococcal spondylodiscitis infections, 4 cases (67%) occurred following posterior spinal surgery. Therefore, this infection could be relatively commonly introduced as a postoperative surgical site infection.

Our findings highlight the sensitivity and specificity of DNA-based diagnostic methods and their contribution to the identification of bacterial pathogens in spondylodiscitis. Molecular analysis can be regarded as a useful adjunct to culture rather than a substitute for it. It can confirm culture results when a rare or atypical pathogenic agent is found, exclude an intercurrent sample contamination, and rapidly recognize non-growing or fastidious pathogens.

In conclusion, based on the findings of our study, patients with lower back pain and tenderness followed by systemic symptoms such as low grade fever with a rise in hematological inflammatory markers should be suspected of having pyogenic spondylodiscitis. In addition, microbiological and molecular methods and MRI proved to be vital components for the diagnosis of pyogenic spondylodiscitis. These findings may be further enhanced by the clinical application of *16S rRNA* sequencing as a diagnostic tool for pyogenic spondylodiscitis. The rapid turnaround time of *16S rRNA* PCR and sequencing submission and results are likely to decrease the time to diagnosis and improve the therapeutic management of these infections, as appropriate antibiotic therapy should ideally be initiated after a definitive etiological diagnosis is made. Although *S. aureus* and *M. tuberculosis* were the most common causes of spinal infection, other infectious agents should also be considered.

## Ethics statement

The initial proposal of the work was discussed and approved on session dated March 21th, 2014, in the University high research and ethics combined committee and has been double approved in Infectious and Tropical Diseases Research Center and was financially supported by Research affairs (OG.92137), Ahvaz Jundishapur University of Medical Sciences, Ahvaz, Iran. Patients before the survey, completed consent form to participate in the study. There was not any additional considerations.

## Author contributions

Molecular genetic studies, designing, and writing of the manuscript carried out by AS, AK, and HG also participated in the primers sequence alignment and drafted the manuscript. HG, MH, RN, AM, AT, MC, RR, MH, and SA carried out specimen collection, phonotypical and molecular methods and primers sequence alignment. Also all authors read and approved the final manuscript.

### Conflict of interest statement

The authors declare that the research was conducted in the absence of any commercial or financial relationships that could be construed as a potential conflict of interest. The reviewer EL and handling Editor declared their shared affiliation and the handling Editor states that the process nevertheless met the standards of a fair and objective review.
